# Medium-Term Effect of Treatment with Intra-Articular Injection of Sodium Hyaluronate, Betamethasone and Platelet-Rich Plasma in Patients with Temporomandibular Arthralgia: A Retrospective Cohort Study

**DOI:** 10.3390/life12111739

**Published:** 2022-10-29

**Authors:** Bruno Macedo de Sousa, Antonio López-Valverde, Francisco Caramelo, María João Rodrigues, Nansi López-Valverde

**Affiliations:** 1 Institute for Occlusion and Orofacial Pain, Faculty of Medicine, University of Coimbra, Polo I-Edifício Central Rua Larga, 3004-504 Coimbra, Portugal; 2 Department of Surgery, University of Salamanca, Instituto de Investigación Biomédica de Salamanca (IBSAL), P.º de San Vicente, 58-182, 37007 Salamanca, Spain; 3 Laboratory of Biostatistics and Medical Informatics, Institute for Clinical and Biomedical Research (iCBR), School of Medicine, University of Coimbra, Polo 3, Azinhaga de Santa Comba, 3000-548 Coimbra, Portugal; 4 Department of Medicine and Medical Specialties, Faculty of Health Sciences, Universidad Alcalá de Henares, 28871 Madrid, Spain; 5 Instituto de Investigación Biomédica de Salamanca (IBSAL), Avda. Alfonso X El Sabio S/N., 37007 Salamanca, Spain

**Keywords:** temporomandibular disorders, arthralgia, sodium hyaluronate, betamethasone, platelet-rich plasma

## Abstract

**Simple Summary:**

Temporomandibular disorders are a major public health problem affecting approximately 10% of the population, currently the second most common musculoskeletal condition after chronic low back pain, and causing disability and pain in patients who suffer from them, limiting the individual’s daily activities and quality of life. Intra-articular injections have been proposed as a specific treatment for joint inflammation and degeneration and have been shown to increase mouth opening and decrease pain associated with these disorders, although long-term follow-up is scarce in the literature. Our study compared the efficacy on TMJ pain of intra-articular injections of betamethasone, sodium hyaluronate and platelet-rich plasma in a sample of 114 patients, with a three-year follow-up, and found that both platelet-rich plasma and sodium hyaluronate led to significant pain-free time after treatment; betamethasone was less effective.

**Abstract:**

Temporomandibular joint disorders are associated with pain and reduced jaw mobility. The aim of this study was to compare the long-term effect on pain of intra-articular TMJ injections of betamethasone, sodium hyaluronate and platelet-rich plasma. The sample was made up of 114 patients, who were randomly distributed into three groups at least three years ago and who achieved a total remission of pain after treatment. We found that the median number of months without pain was, according to each group, as follows: platelet-rich plasma: 33; sodium hyaluronate: 28; betamethasone: 19. Both platelet-rich plasma and sodium hyaluronate lead to significant pain-free time after treatment; when we compare bethametasone with the two other substances, it proved to be very ineffective.

## 1. Introduction

Temporomandibular joint disorder (TMD) is a significant public health disorder affecting between 5% and 12% of the population on average. After persistent low back pain, TMD is the second most prevalent musculoskeletal disorder causing discomfort and impairment. [1] The Diagnostic Criteria (DC) for TMD is intended for use in any clinical setting and supports the full range of diagnostic activities from screening to definitive evaluation and diagnosis. Using this, TMD could be divided into muscle disorders (including myofascial pain with or without referral and with or without mouth opening limitation) and intra-articular disorders (including disc displacement with or without reduction and mouth opening limitation, arthralgia, arthritis and degenerative joint disorders) [2].

The most common clinical manifestations are pain, mouth opening limitation, muscle or joint tenderness on palpation, changes of mandibular movements, joint sounds and otologic complaints like tinnitus or vertigo [3].

Temporomandibular joint disorders (TMJDs) is a collective term that refers to a range of pathologies affecting the jaw joints and associated structures, resulting in internal joint space dysregulation, bony changes and degenerative pathologies. TMJDs are characterized by pain, joint noise, limited range of motion, impaired jaw function, deviation when opening and closing the mouth and open locking. They are a very common pathological agent affecting about 10% of the population, and are twice as frequent in women than in men [4,5,6].

In clinical practice, different treatments have been proposed to alleviate joint pain and disc displacement, including conservative therapies such as non-steroidal anti-inflammatory drugs (NSAIDs), mandibular rest, splints and physiotherapy, as well as surgical procedures such as arthrocentesis, disc repositioning or discectomy in patients who do not respond to conservative treatments. A conservative approach should always be taken primarily [7,8].

In 1953, Horton et al. were the first to propose intra-articular administration of corticosteroids (CS) for the treatment of temporomandibular joint osteoarthritis (TMJO) [9]. Since then, several investigators have confirmed that intra-articular injections of hydrocortisone, prednisolone and betamethasone reduce joint pain. It is commonly accepted that therapy with intra-articular corticosteroid injections is a procedure used mainly in patients who have not achieved satisfactory results with other less invasive approaches [10,11].

Sodium hyaluronate (SH), a hyaluronic acid (HA) derivative, is a material of high molecular density and high viscosity, essential for joint lubrication and cartilage protection, which would reduce granulation tissue formation and intra-articular adhesions, having been proposed as an additional therapy with similar therapeutic effects [12,13]. There is speculation as to whether SH not only acts as a viscosupplement in reducing mechanical friction, but also plays a role in inflammatory mediators in the osteoarthritic phase, controlling the proteolytic activation of plasminogen activator and preventing the release of proinflammatory mediators such as IL-1b or the indirect activation of metalloproteinases [14].

Platelet-rich plasma (PRP) is an autologous blood product obtained by peripheral venipuncture and subsequent centrifugation. The concentrated plasma product contains a high concentration of platelets, which play important roles in tissue homeostasis and control of inflammation, as well as in inhibition of chondrocyte apoptosis, bone and vascular remodeling and collagen synthesis. Certain studies have compared the clinical results of intra-articular injection of PRP with other conservative treatment methods such as corticosteroid injection [15,16,17].

Platelets are enucleated cells derived from megakaryocytes [18]. When platelets are activated, the growth factors contained in the α-granules react in a localized and specific manner. These growth factors, in addition to coagulation factors, cytokines, chemokines and other proteins contained in platelets, have been shown to induce proliferation of chondrocytes and chondrogenic mesenchymal stem cells (MSCs), which promote the secretion of cartilaginous matrix from chondrocytes and reduce the catabolic effects of proinflammatory cytokines [19,20,21,22,23].

The aim of this study was to analyze how long a patient could remain pain free after being treated with one of three intra-articular infiltrations—betamethasone, SH or PRP—as a continuation of a previous study [23] that assessed the short-term effect of these three infiltrations. From that research we selected the patients who achieved complete pain remission after treatment and followed them for three years. No patient dropped out. 

## 2. Materials and Methods

### 2.1. Patients

A sample of 114 patients who were diagnosed with TMJ arthralgia was selected, according to the original version of the diagnostic criteria for TPMJDs, such as local pain modified with movement and pain on palpation, and who were treated with intra-articular injection of PRP, SH or betamethasone, achieving no pain after treatment and who were followed up for at least 3 years, in order to study the duration of the treatment effect. The patients were recruited and treated, after the conservative approach showed no effect, in consultations within the framework of the Occlusal Rehabilitation Course of the University of Coimbra, organized by the Faculty of Medicine. All patients agreed to participate in the research and signed the consent forms. This study was conducted in accordance with the Declaration of Helsinki and was approved by the ethics committee of the Faculty of Medicine of the University of Coimbra (Coimbra, Portugal). This study was approved on 25 June 2017 by the institutional review board (IRB 06-2017-096). No patient has dropped out from the study.

### 2.2. Inclusion and Exclusion Criteria

Inclusion: All patients with a clinical history of more than 6 months of TMJP that is modified by mandibular movement in function or parafunction; pain present on clinical examination on opening, lateral movements or palpation; and without previous effective treatment. All patients included in this study have achieved, after treatment, a grade 0 according to the Visual Analogic Scale (VAS).

Exclusion: patients who had received effective previous treatment for TMJ dysfunction; patients suffering from any rheumatic pathology such as rheumatoid arthritis or psoriatic arthritis (including juvenile arthritis); patients undergoing hypnosis; pregnant or breastfeeding women; patients under 18 years of age; and patients who had partial or no relief of the pain condition and had to receive another treatment. 

Prior to treatment, patients were randomly assigned to three groups: patients receiving an intra-articular injection of PRP, betamethasone or SH.

### 2.3. Treatments

The following protocol was followed: after disinfection of the preauricular area, patients were injected with 1 mL of articaine (40 mg/mL) and adrenaline (10 μg/mL). A 23-gauge needle was used to inject 1 mL of betamethasone (Diprofos Depot^®^, Schering-Plough Labo, Heist-Op-Den-Berg Belgium 14 mg/2 mL) or 1 mL of SH (Hyalart^®^, Grunenthal GmbH, Achen, Germany, 20 mg/2 mL). The exact puncture point was determined by tracing the canthus-tragus line and measuring 10 mm from the tragus and 2 mm below the line. The zygomatic arch was palpated, and patients were asked to open their mouth to move the condyle forward. The position of the needle was from outside to inside, top to bottom, and back to front. Patients were informed that they might experience discomfort in the region. No analgesic or anti-inflammatory drugs were prescribed. During the whole process, all patients were always followed by the same professional. Alternatively, after signing the informed consent, patients were randomly assigned to the corresponding treatment group. Each patient’s treatment was assigned by means of a randomization list automatically generated before the start of the study in which the treatment approach was determined. 

In the PRP group, injections were preceded by drawing the patient’s peripheral blood from the cubital vein into a glass tube with sodium citrate as anticoagulant. After mixing the blood with the citrate, with rotating movements, the tubes were centrifuged at 3200 rpm for 12 min. After careful aspiration of the platelet-rich plasma into a syringe, 2 mL of PRP was injected into the TMJ following the procedure described above for betamethasone and sodium hyaluronate injections.

### 2.4. Statistics

The statistical analysis focused on the description of the data using absolute and relative frequencies for qualitative and mean variables, minimum and maximum standard deviation for quantitative variables. Sex was evaluated between the different groups by Fisher’s exact test and age by the Kruskal-Wallis test. Pain-free time was compared between the groups by means of the ANCOVA test in which age was used as a covariate and sex as an additional factor. Weibull analysis was also performed to determine the median time without pain. The assumption of normality was evaluated by the Shapiro-Wilk test.

In the statistical analysis, a significance level of 0.05 was considered, having been performed on the IBM^®^ SPSS^®^ v26 platform and on MATLAB (R2019b).

## 3. Results

The sample of this study was composed of 114 individuals, 27 men and 87 women randomly distributed in 3 groups.

In the betamethasone group there were 8 men and 26 women, the mean age was 41.2 years, the youngest being 18 years and the oldest 66 years.

In the SH group the gender distribution was 9 men and 30 women. With respect to age, the minimum was also 18 years, while the maximum was 65 years.

Finally, in the PRP group, composed of 10 men and 31 women, the age range was 18 to 66 years, with a mean of 37.6.

The maximum number of months of follow-up of the patients was 36 months (3 years). It is important to mention that 33 of the 41 patients treated with PRP were permanently pain-free. The same result was obtained by 19 of 39 patients treated with SH. The worst result was observed in the betamethasone group, in which only 9 of the 34 patients were able to remain pain-free for the entire 36-month period.

The following table shows the statistics of age and sex in the three groups defined by the substance injected (Table 1).

**Figure 1:**
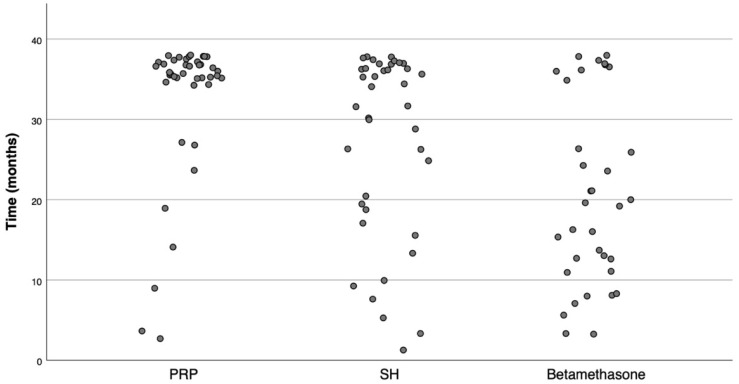
Distribution of pain-free times.

Statistics on pain-free time are presented in Table 2. Figure 1 shows the distribution of pain-free times for the three groups. The results emphasize the contrast between betamethasone and the two other substances.

Statistically significant differences are observed (*p* = 0.005) between the groups in terms of administered substances, but neither age (*p* = 0.827) nor sex (*p* = 0.403) have an impact on pain-free time. To compare the groups among themselves, the Dunn-Sidak post-hoc test was chosen, whose results were:

- PRP vs. SH: *p* = 0.048
- PRP vs. Betamethasone: *p* < 0.001
- SH vs. Betamethasone: *p* = 0.069

**Figure 2:**
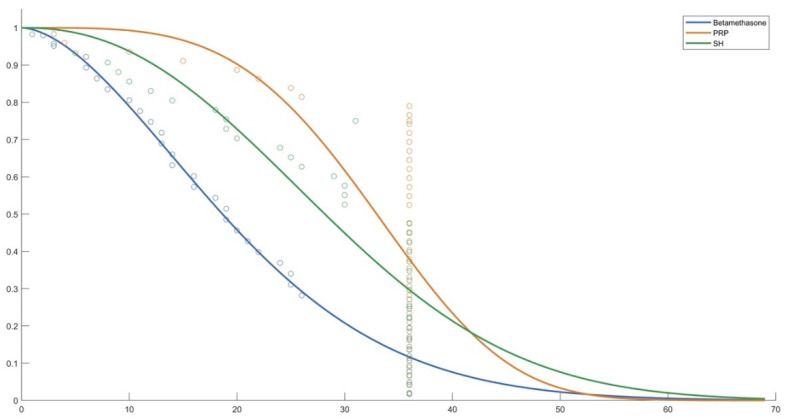
Probability curve for the three substances used.

**Figure 3:**
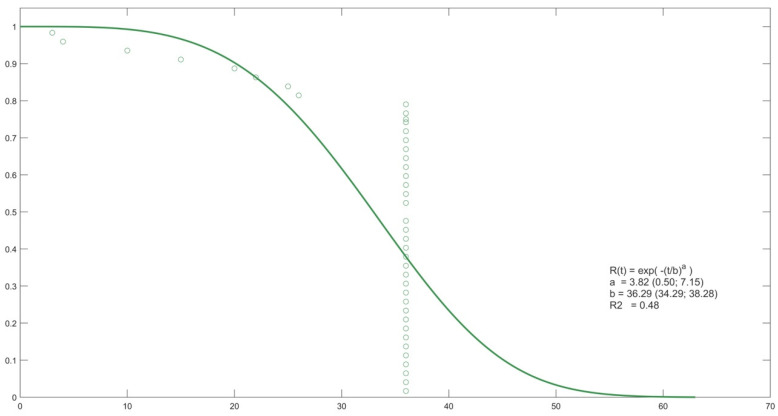
Probability curve for PRP.

**Figure 4:**
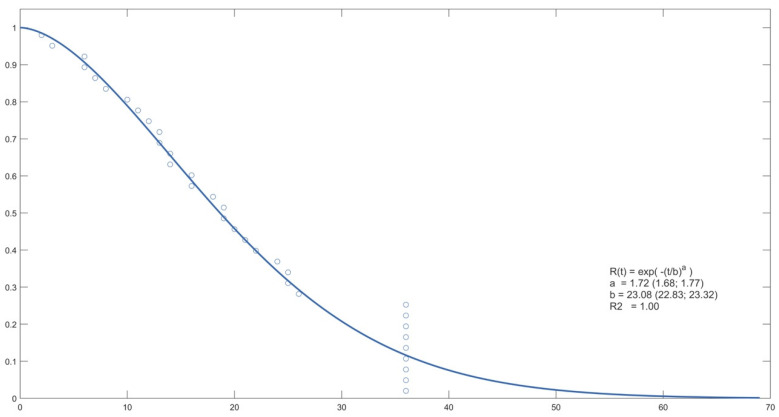
Probability curve for SH.

The following figures show the probability curve of pain-free time obtained by Weibull analysis (Figure 2, Figure 3 and Figure 4).

## 4. Discussion

The TMJ is the only double joint in which two separate joints must move in a coordinated manner. if an imbalance is caused it can result in pathological states of one or both sides of the joint. In modern society, increasingly stressful eating habits and lifestyles make an increasing percentage of the population susceptible to TMD [24]. Intra-articular injection therapy is used particularly in patients who do not obtain favorable results with other conservative methods. The aim of this study was to analyze the effects of three intra-articular infiltrations (betamethasone, SH and PRP) on TMJ joint pain following the same protocol of a previous randomized clinical trial that evaluated the short-term effect of these three substances [23].

Several previous studies have demonstrated the efficacy of intra-articular infiltrations of betamethasone, HA and PRP [4,6,13,25,26,27,28,29,30,31,32], despite the fact that most research relates infiltrations to more invasive procedures, such as arthrocentesis, which is especially useful in TMJDs and can be performed as an outpatient treatment under local anesthesia. Currently, arthrocentesis generally uses two needles, one for serum inflow and one for outflow, although techniques using a single needle have been described [33], but there is no evidence of superiority of the latter [34].

Moreover, it should be noted that this type of treatment does not require the use of complex or expensive equipment [35].

Most studies point to the short-term benefits of this type of treatment, with a paucity of long-term studies [36]. Intra-articular betamethasone injections have been the most widely followed [37,38].

Gokçe et al. [11], in a study of 60 patients, compared clinically and radiologically the effects of PRP, HA and corticosteroid injections in osteoarthritis of the TMJ, finding no significant (short-term) differences in the assessment of crepitus or pain. Jüni et al. [39], in a Cochrane review of 27 trials with 1767 participants, drew attention to the use of intra-articular corticosteroids, their benefits and safety, highlighting the uncertainty of their clinical benefits, together with the low methodological quality of the included RCTs. They further highlighted the lack of benefit after 6 months of follow-up, coinciding with our study, in which both PRP and SH led to significant time without pain after treatment, while betamethasone was less effective.

Comparative randomized studies of intra-articular injections of HA (SH) or cortico-steroids (betamethasone) into the TMJ have not demonstrated substantial differences between the two therapies. Follow-ups of one or two years reported sustained and substantial reductions in TMJ symptoms and increased joint mobility [28,38]; Tanaka et al. [40] used HA injections to increase synovial fluid viscosity and decrease inflammatory markers, but despite short-term clinical improvement, their research did not provide evidence to support the hypothesis that HA can modify the arthritic environment and reverse or slow long-term cartilage deterioration. Hegab et al. demonstrate that HA injections have similar effects to PRP injections at mid-term follow-up; however, PRP allows for better results in long-term follow-up, with no recurrence of joint pain and sound at 12 months [41]; Al-Delayme et al. reported the efficacy of PRP injection as a primary treatment for nonreducing disc displacement but limited the period of clinical benefit to 6 months, after which an additional injection may be necessary [42].

PRP has an effect on chondrogenic differentiation and tissue remodeling. It has been studied more in other joints than in the TMJ [43]. Given the anti-inflammatory potential of PRP, several studies and meta-analyses have explored the curative effect of intra-articular PRP injection in the treatment of patients with joint pathology [44,45,46,47].

A study by Joshi Jubert et al. concluded that PRP was effective in relieving pain and improving TMJ function, although the effects of PRP were comparable with the effects of corticosteroids in patients with advanced stage osteoarthritis (OA) [48]. Other studies argued that a single injection of PRP relieved pain and symptoms better than corticosteroids, noting that PRP produced beneficial effects in the treatment of OA, up to 12 months after intra-articular injection [49,50]. Some research has indicated that the benefits of intra-articular PRP injections in the reduction and relief of joint pain may be due to its effect on inflammatory mediators, increasing jaw dynamics and masticatory performance [27,51,52].

In general, the results of the different studies on the use of betamethasone, SH and PRP in the treatment of TMJ diseases coincide with the results of our studies. Our first study [23] compared the effectiveness of intra-articular injections with SH, PRP and betamethasone in the short term (1 week, 1 month and 6 months). In that study, betamethasone and SH intra-articular injections showed the greatest decrease in pain in the first week, but at 6 months, PRP showed the best results. This second, long-term investigation confirmed our first results.

## 5. Conclusions

In our study, both PRP and SH led to significant pain-free time after treatment; betamethasone was less effective. However, more rigorous randomized controlled trials with long-term follow-up are needed.

## Figures and Tables

**Table 1:** Age and sex statistics in the three groups.

	PRP (41)	SH (39)	Betamethasone (34)A	*p*
sex (M/F)	10/31 (24.4%/75.6%)	9/30 (23.1%/76.9%)	8/26 (23.5%/76.5%)	1.000 ^§^
age $\bar{x}\pm sd$ (min/max)	37.6 ± 15.0 (18/66)	39.1 ± 13.1 (18/65)	41.2 ± 15.3 (18/66)	0.570 ^£^

^§^ Fisher; ^£^ Kruskal-Wallis exact test.

**Table 2:** Pain-free time for PRP, SH and betamethasone.

	PRP (41)	SH (39)	Betamethasone (34)A	*p*
time $\bar{x}\pm sd$ (min/max)	32.0 ± 9.0 (3/36)	26.8 ± 11.4 (1/36)	20.4 ± 11.2 (2/36)	0.005 ^#^

^#^ ANCOVA (age: *p* = 0.827; gender: *p* = 0.403).

## Data Availability

Faculty of Medicine, University of Coimbra Database.

## Abbreviations

TMJDs	Temporomandibular joint disorders
NSAIDs	Non-steroidal anti-inflammatory drugs
CS	Corticosteroids
TMJO	Temporomandibular joint osteoarthritis
SH	Sodium hyaluronate
HA	Hyaluronic acid
PRP	Platelet-rich plasma
MSCs	Mesenchymal stem cells
VSA	Visual Analogic Scale
